# Outcomes of Gallic Acid on Alternariol Induced Cyto-Morphic and Genotoxic In Vivo Changes in Parotid Gland: 4-HNE Incorporated

**DOI:** 10.3390/biomedicines7040084

**Published:** 2019-10-27

**Authors:** Mai A. Samak, Ahmed Elshatory, Eman M. Mohamed

**Affiliations:** 1 Department of Histology and Cell Biology, Faculty of Medicine, Zagazig University, Zagazig 44519, Egypt; emanmosallam79@gmail.com; 2 Department of Forensic Medicine and Clinical Toxicology, Faculty of Medicine, Cairo University, Cairo 11865, Egypt; ahmedelshatory@hotmail.com

**Keywords:** alternariol, mycotoxin, parotid gland, gallic acid, 4-HNE

## Abstract

*Alternaria* toxins are emerging mycotoxins that gained considerable interest with increasing evidence of their existence and toxicological properties. There is limited research and insufficient data about their in vivo hazardous effects. We designed this study to evaluate histopathological and genotoxic in vivo impacts of alternariol (AOH) on the parotid gland as well as to assess the competency of gallic acid (GA) in reversing these effects. Forty healthy adult male Wister rats were utilized and assigned equally on control, GA, alternariol and AOH+ gallic treated groups. Parotid gland samples from experimental groups were collected and then examined for histopathological, ultrastructural and immunohistochemical examination for 4-hydroxynonenal “4-HNE as lipid peroxidation marker” as well as Comet assay for DNA damage. Additionally, parotid tissue homogenates were tested for catalase “CAT”, superoxide dismutase “SOD” and malondialdehyde “MDA” levels. Our data proved that alternariol produced various histopathological and ultrastructural alterations of parotid acini as well as significant DNA damage, significant reduction of CAT and SOD enzymatic activity and significant boosting of 4-HNE immunohistochemical expression and MDA levels as compared to control group. On the other hand, gallic acid administration almost restored histological and ultrastructural parotid architecture, 4-HNE immune-expression and biochemical levels. Ultimately, we demonstrated alternariol-induced histopathological and genotoxic alterations on parotid gland as well as the competency of gallic acid in reversing these effects.

## 1. Introduction

Fungi are responsible for producing many toxic metabolites, especially mycotoxins. These fungi mainly belong to *Aspergillus, Penicillium and Alternaria* genera [1]. Contagion of agricultural crops by such fungi causes plant diseases and production of several mycotoxins as aflatoxins by aspergillus [2], ochratoxin by *penicillium* [3] and fumonisins, trichothecenes, zearalenone by *Fusarium* [4]. Mycotoxins occur naturally in cereals, fruits and vegetables, thus, they can appear in the food chain as a result of fungal infection of crops, either when they are directly consumed by humans or when they are used as livestock feed [5]. *Alternaria alternata* (black rot) are common plant pathogens with an observable ability to adapt to surrounding environmental conditions. They are found in semidry and humid regions. They can tolerate lower temperatures; therefore, food refrigerated during storage and transportation can also be contaminated [6]. They produce more than 70 myco and phytotoxins; the most toxic are alternariol (AOH), L-tenuazonic acid (TeA), tentoxin (TEN), alternariol monomethylether (AME) and altenuene (ALT) [7].

Alternariol is an unavoidable contaminant of fruit, vegetables, such as bell peppers, apple, mandarin and tomato, and processed fruit products such as juices [8]. Moreover, it has been discovered in cereals, grain [9] and in nuts and pistachios [10]. Random samples of agricultural foodstuffs in Europe have been claimed 31% to be contaminated by alternariol. Alternariol concentrations diverge from 6.3 to 1840 µg/kg [11]. However, alternariol concentrations in stored tomato for 4 weeks at normal room temperature were elevated to 50 mg/kg [8]. In sunflower seeds, this may reach 1840 μg/kg and in cereals 4310 μg/kg. Until now, there has been no policy for AOH contamination of food and feed. Thus, the dietary exposure has to be low (1.9–39 ng/kg/bw/day) [11,12].

In vivo and non-genotoxic effects of Alternaria mycotoxins were not sufficiently estimated; however, recent studies reported that they act as endocrine disruptors. Alternariol is a diphenolic compound with structural similarities to natural or synthetic oestrogens. Therefore, it behaves as a weak estrogenic mycotoxin that also has the ability to interfere with the steroidogenesis pathway or block estrogen receptors [13]. On the other hand, estradiol and progesterone production levels in human adrenocortical carcinoma cells increased in response to alternariol exposure [14].

Nowadays, antioxidants derived from natural sources, especially plants, attracted notable interest in scavenging reactive oxygen species (ROS). These antioxidants include flavonoids, anthocyanins and phenolic compounds [15]. Gallic acid (3,4,5-trihydroxybenzoic acid), a naturally occurring versatile triphenolic compound found in a wide plethora of plants and herbs such as blueberries, walnuts, apples, flax seed, and also in spices (sumac). It has reported antibacterial and antifungal properties against a wide range of pathogens, including Escherichia coli, Staphylococcus aureus and Aspergillus [16]. It also proved to express therapeutic effects such as anti-allergic, anti-inflammatory [17], anti-mutagenic and anti-carcinogenic [18,19]. In addition, gallic acid was found to ameliorate impaired glucose and lipid homeostasis in nonalcoholic fatty liver disease [20]. Moreover, Hsu and Yen [21] reported that gallic acid modified high fat diet-induced dyslipidaemia, hepatosteatosis and oxidative stress. Furthermore, Sen et al. [22] proved antioxidant and antiulcerogenic potentials of gallic acid in gastric ulcer.

Only limited data about alternariol in vivo toxic effects are available; however, it has been implicated in an elevated incidence of esophageal carcinogenesis [11]. Meanwhile, no further experimental studies have been performed to clarify other possible risks. Hence, we designed this study to evaluate histopathological and genotoxic in vivo impacts of alternariol on parotid gland, and to assess the competency of gallic acid in reversing these effects.

## 2. Materials and Methods

### 2.1. Chemicals

Alternariol (AOH): 3,7,9-Trihydroxy-1-methyl-6H-dibenzopyran-6-one, as Empirical Formula from Alternaria sp. (White to Yellow powder, CAS No: 641-38-3). It was purchased from Sigma-Aldrich (St. Louis, MO, USA).

Gallic acid: 3,4,5-Trihydroxybenzoic acid (White powder, CAS No: 149-91-7). It was purchased from Sigma-Aldrich (St. Louis, MO, USA).

### 2.2. Experimental Animals

Forty Wistar rats; adult (7 to 9-week age); male; weighing 200–250 g were obtained from the Animal House of the Faculty of Medicine, Zagazig University, Egypt. These animals were placed in plastic cages under normal laboratory conditions with suitable humidity and controlled photoperiod of 12 h-dark and light. They were allowed ad libitum access to food and water. All procedures were done according to institutional guidelines for the use of experimental animals and approved by IACUC, Zagazig University (Zagazig, Egypt). All rats received humane care in compliance with the Ethical Committee of Zagazig University and in accordance with the NIH Guidelines for the Care and Use of Laboratory Animals (March 2019, No. ZU-IACUC/3/F/66/2019).

### 2.3. Experimental Procedure

After 1-week acclimation, rats were parted randomly into four groups (10 rats each):

**Group I (control group)**: continued to drink tap water and standardized diet.

**Group II (GA group):** received 50 mg/kg gallic acid dissolved in 1 mL saline solution by oral gavage daily for 14 days [23].

**Group III (Alternariol group)**: received single dose of Alternariol “AOH” mycotoxin 10 mg/kg dissolved in ethanol and sunflower seed oil by oral gavage [24].

**Group IV (AOH+ gallic acid treated group):** received single dose of Alternariol 10 mg/kg, then treated with 50 mg/kg gallic acid by oral gavage daily for 14 days.

At the end of experiment: rats were sacrificed by intraperitoneal thiopental injection 50 mg/kg [25], parotid specimens were cut; parts of them for histopathological preparation; parts for comet assay and others were frozen immediately and stored at –80 °C until the preparation of tissue homogenates for biochemical and molecular analyses.

### 2.4. Histopathological Study

#### 2.4.1. Haematoxylin and Eosin (H&E) Stain

Specimens for light microscopy were fixed in 10% saline formalin and processed to prepare 5-μm-thick paraffin sections for H&E stain [26].

#### 2.4.2. Immunohistochemical Study

Avidin biotin complex (ABC) method (Dako ARK™, Peroxidase, Code No. K3954, Dako, Glostrup, Denmark) is the method used for Immunohistochemical staining of 4-hydroxynonenal (4-HNE) as a lipid peroxidation marker. Removal of wax and hydration of paraffin sections were the beginning points of the procedure. Antigen recovery was then performed by using citrate buffer. Tissues block was done by bovine serum albumin. Then, sections were incubated with the specific primary antibody overnight (4 °C): anti-4-hydroxynonenal (4-HNE) antibody (mouse monoclonal antibody; No. ab48506; dilution 1/200; Abcam, Cambridge, UK). Recognition was performed by secondary antibodies and labeled horseradish peroxidase, after that; colorimetric detection by 3, 3′-diaminobenzidine (DAB). Tissues were counterstained with hematoxylin. Negative control sections were put in phosphate-buffered saline instead of the primary antibody. Under light microscopes; the brown-color indicated the antigen site [27].

### 2.5. Ultrastructure Study

Fixation of the specimens was done by phosphate-buffered glutaraldehyde (pH 7.4), and post fixation by 1% osmium tetroxide at 4 °C; then, dehydration and embedding in epoxy resin occurred. Cutting by (Leica ultra-cut UCT), staining was performed by uranyl acetate and lead citrate [28]. Examination and photography using (JEOL JEM 1010 transmission electron microscope; Jeol Ltd., Tokyo, Japan) in the Regional Center of Mycology and Biotechnology (RCMB), Al-Azhar University, Egypt.

### 2.6. Alkaline Single Cell Gel Electrophoresis (Comet Assay)

Parotid specimens from each rat were taken and kept in physiological saline (0.9% NaCl) at −20°C and 10% dimethyl sulfoxide (DMSO) for cryopreservation until used for the comet assay to determine the extent of DNA damage [29].

The Animal Reproductive Research Institute (ARRI) of Agricultural Research Centre of Ministry of Agriculture and Land Reclamation, Egypt was the place where the comet assay was done. pH condition was >13 according to the method of [30], which is shortened as follows:

Crushed samples of 0.5 g each were put in 1 mL ice-cold PBS. The formed cell suspension (100 μL) was mixed with 600 μL of low-melting agarose (0.8% in PBS). Spreading on pre-coated slides was performed. Then, the slides were put in lyses buffer (0.045 M TBE, pH 8.4, containing 2.5% SDS). After that, they were exposed to electrophoresis containing the same TBE “Tris/Borate/EDTA” buffer, but devoid of SDS “sodium dodecyl sulfate”. The slides then were stained by ethidium bromide 20 μg/mL at 4 °C. DNA fragments of 100 cells for each dose level were examined with a fluorescence microscope using 20× objective lens. Measuring tail lengths was done from the center of the nucleus to the end of the tail, with a 40× increase for the count, after which the comet size was measured.

We used Comet 5 image analysis software for the quantitation of SCGE data. This was developed by Kinetic Imaging, Ltd. (Liverpool, UK) linked to a CCD camera. It measures the extent of DNA damage in the cells (50 to 100 randomly selected cells are analyzed per sample) and tail moment. 

### 2.7. Biochemical Analysis of Tissue Antioxidant Enzymes

Homogenates of the tissues were placed in cold ice 0,1 M Tris–HCl buffer (pH 7.4). These were centrifuged at 8000× *g* for 30 min at 4 °C to get rid of the cell debris. The antioxidant enzymes Catalase (CAT) and Superoxide dismutase (SOD), as well as malondialdehyde (MDA), the lipid peroxidation marker, were caught in the supernatant by the aid of commercial kits (Bio Diagnostic Company, Dokki, Giza, Egypt). The results were expressed as U/mg protein.

### 2.8. Morphometric Study

The data were investigated by Leica QWin 500 software using digital camera linked to an optical microscope (Olympus, Tokyo, Japan). Area percent/20 mm^2^ frames at 400× magnification for positive (4-HNE) immune reactions was performed. Ten non-overlapping fields were randomly selected and investigated from each rat in each group by examiner who was ignorant about the experiment.

### 2.9. Statistical Analysis

SPSS statistical software version 20 was used to analyze the data. Values were expressed as means ± standard error of means (SEM). ANOVA test followed by Tukey’s post-hoc test was used. The probability values (*p*) less than 0.05 were thought to be significant and it will be highly significant with *p* values less than 0.001.

## 3. Results

### 3.1. Histopathological Results

Examinations of group I and II revealed similar results. Only morphological results of group I were presented.

#### 3.1.1. Light Microscope Results

**Figure 1:**
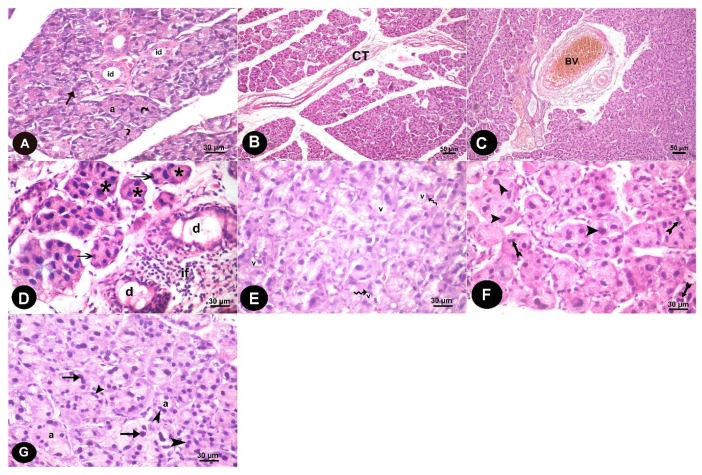
Haematoxylin and Eosin stained sections in parotid tissue of albino rats of the study groups show: Control group: (**A**) The lobular pattern of parotid glandular parenchyma with its serous acini (a) that are lined by pyramidal-shaped cells with rounded basal nuclei (arrow). The interlobular ducts (id) are lined with cubical epithelium (curved arrow). Alternariol group: (**B**) Thick connective tissue septa containing many fibers (CT) are observed between lobes. (**C**) large congested blood vessels (Bv) appeared in the septa. (**D**) disorganized acini with a reduction or disappearance of the central lumen (*). Dark pyknotic nuclei appeared in the epithelial lining the acini (arrows). Many dilated interlobular ducts (d) and cellular infiltration (if) are also seen. (**E**) The cytoplasm of the acinar cells showed variable sized vacuoles (v) that displace the nuclei more peripherally (zigzag arrow). (**F**) some acini have dividing nuclei indicating mitosis (arrow head) and others contain large sized nuclei (crossed arrow). AOH+ gallic acid group: (**G**) Most of the acini exhibit well-demarcated edges (a). However, some acini had dark nuclei, while others still appeared to have dividing nuclei indicating mitosis (arrow head). Some cells appeared with few vacuoles (v).

Histological results of H&E-stained sections of parotid gland of control group revealed that the glandular parenchyma was arranged in typical lobular structures containing serous acini and ducts. These acini had central lumen and were seen lined by pyramidal cells. The lining epithelium of intralobular ducts was cuboidal (Figure 1A). The alternariol treated group displayed widely separated acini by thick connective tissue and congested blood vessels (Figure 1B,C). Acini appeared disorganized with a reduction or disappearance of the central lumen, their epithelial lining revealed dark pyknotic nuclei. Many dilated interlobular ducts and cellular infiltration also appeared (Figure 1D). Many vacuoles appeared in the acinar cells, cytoplasm and displaced the nuclei peripherally (Figure 1E). Some acini have dividing nuclei indicating mitosis and others contain large sized nuclei (Figure 1F). The parotid gland of the recovery group showed acini with well-demarcated edges. However, some acini had dark nuclei, while others still appeared to have dividing nuclei, indicating mitosis (Figure 1G).

#### 3.1.2. Immunohistochemical Results

**Figure 2:**
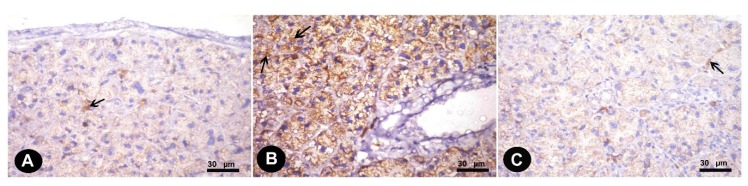
Immunohistochemically stained sections in the parotid of albino rats of different groups. Cytoplasmic immune reaction for 4-hydroxynonenal (4-HNE) (**A**) Control group (weak positive reaction “arrow”) (**B**) Alternariol group (stronger positive reaction “arrow”) (**C**) AOH+ gallic treated group (moderate reaction “arrow”).

Immunohistochemically stained sections for (4-HNE) antibodies in the parotid of the control group revealed weak positive cytoplasmic reaction in parotid acinar cells (Figure 2A). The alternariol treated group showed stronger positive immunoreactions (Figure 2B); however, gallic acid treated group revealed moderate immunoreactions compared to treated group (Figure 2C).

### 3.2. Electron Microscope Results

**Figure 3:**
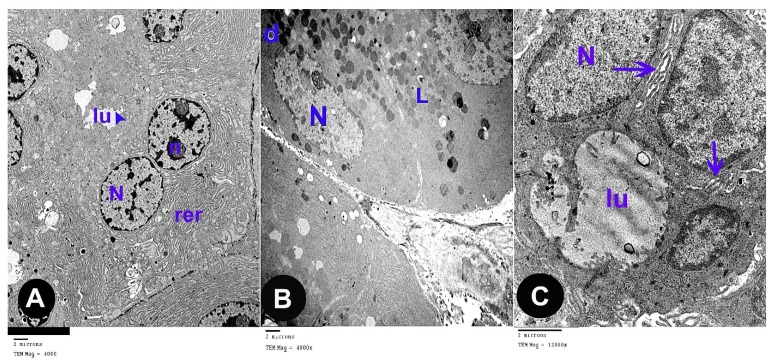
Electron microscope sections in parotid tissue of albino rats of the control group: (**A**) serous acini with pyramidal shaped cells which contained euchromatic rounded nuclei (N), prominent nucleoli (n) and rough endoplasmic reticulum (rer). Microvilli (arrow head) of the acinar cells appeared at the luminal surface (lu). (**B**) Electron-dense granules (d) and electron-lucent granules (L) (**C**) Serous acini with their lumen (lu); they are lined by pyramidal shaped cells with euchromatic nuclei (N) and intact basement membrane with interdigitations (arrow).

The ultra-thin sections of the control group revealed that the parotid gland appeared containing serous acini covered by pyramidal cells holding euchromatic rounded nuclei with apparent nucleoli. Microvilli were projecting to the lumina. Rough endoplasmic reticula were abundant in the cytoplasm (Figure 3A). Electron-dense secretory granules and few electron-lucent granules were observed (Figure 3B). The cells were strictly interdigitated at their lateral borders and enclosed by intact basement membrane. (Figure 3C).

**Figure 4:**
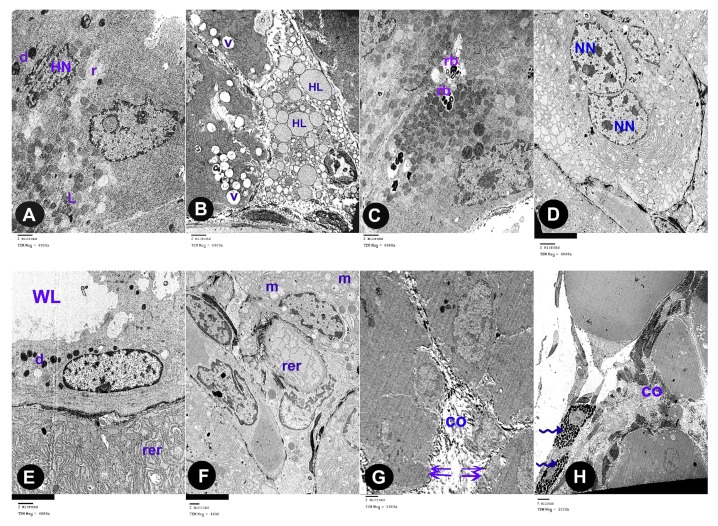
Electron microscope sections in parotid tissue of albino rats of the Alternariol group: (**A**) acinar cells with rarified cytoplasm (r). Heterochromatic irregular shrunken nuclei (HN). Electron dense secretory granules (d), electron lucent granules (L). (**B**) Huge granules (HL), cytoplasmic vacuoles (V). (**C**) Some cells contain residual bodies (rb). (**D**) Other cells contain double nuclei (NN). (**E**) Dilated, irregular, rough endoplasmic reticulum (rer), electron dense secretory granules (d) and wide acinar lumen (WL). (**F**) Deformed mitochondria (m) and dilated, irregular, rough endoplasmic reticulum (rer). (**G**) Widening of intercellular spaces (double arrows) with many collagen fibers (co). (**H**) Mast cells with many secretory granules (zigzag arrow) and collagen fibers (co).

The ultra-thin sections of the parotid gland of AOH group revealed several forms of degeneration in the serous acinar cells in the form of rarefied cytoplasm and cytoplasmic vacuoles, dark heterochromatic irregular shrunken nuclei. Some cells had electron dense secretory granules and others had electron lucent granules, some of them are huge in size (Figure 4A,B). Some cells contain residual bodies (Figure 4C), other cells contain double nuclei (Figure 4D). Some acinar cells had wide lumina, others had dilated irregular rough endoplasmic reticulum, deformed mitochondria and numerous large cytoplasmic vacuoles (Figure 4E,F). Widening of intercellular spaces was seen with many collagen fibers (Figure 4G). Many inflammatory cells as mast cells with many secretory granules were seen (Figure 4H).

**Figure 5:**
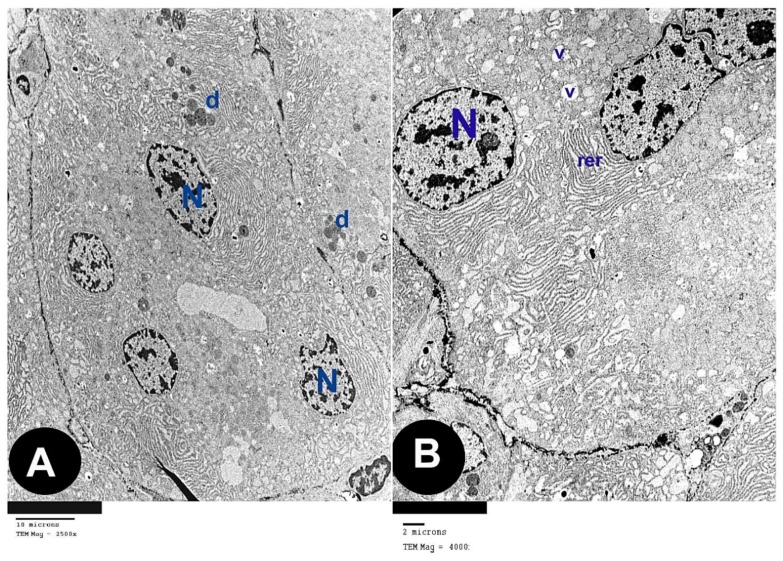
Electron microscope sections in parotid tissue of albino rats of the AOH+ Gallic acid group: (**A**) serous acini are lined with pyramidal-shaped cells with euchromatic nuclei (N) and small, dense granules (d). (**B**) Rough endoplasmic reticulum (rer). Few acinar cells have cytoplasmic vacuoles (v).

The ultra-thin sections of the parotid gland of AOH+ gallic acid group disclosed normal acinar structure covered by pyramidal cells having euchromatic nuclei. Their cytoplasm hold abundant small, dense granules, rough endoplasmic reticulum and few vacuoles (Figure 5A,B).

### 3.3. Results and Statistical Analysis of Comet Assay

**Figure 6:**
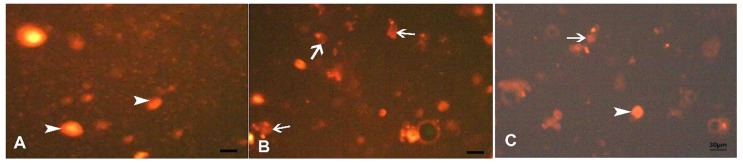
Comet assay of acinar cells of the parotid of adult male albino rats of study groups. (**A**) Control group, normal condensed type nuclei and undamaged cells (arrow head). (**B**) AOH group, abnormal tailed nuclei and damaged cells (arrow). (**C**) AOH+ gallic group, some cells with tailed nuclei (arrow) and other are normal (arrow head).

The present study tested the in vivo genotoxic potential of alternariol in rats using the single cell gel electrophoresis (comet assay). The control specimens revealed normal condensed nuclei and undamaged cells of control group (Figure 6A). Group (III) shows abnormal tailed nuclei and damaged cells (Figure 6B). Group (IV) shows some tailed nuclei together with the undamaged cells (Figure 6C).

### 3.4. Morphometric Results

Our statistically analyzed results for area (%) of positive (4-HNE) immune reactions were summarized in (Table 1).

The parameters used to measure DNA damage in the cells were the following: % of tailed nuclei, tail length (length of DNA migration), tail DNA % (percentage of migrated DNA in the tail) and unit tail moment (correlation between tail length and tail DNA %) (Table 2). Alternariol treated group (II) showed a significant increase in % of tailed nuclei, tail length, tail DNA % and unit tail moment of nuclei of acinar cells compared with the nuclei of both control (I) and gallic acid (IV) groups.

### 3.5. Biochemical Results

Assessment of the activities of CAT, SOD and MDA revealed a significant decrease in group III compared with group I and IV (Table 3).

## 4. Discussion

As *Alternaria* toxins now represent emerging mycotoxins with increasing evidence of their existence and toxicological properties, they gained considerable interest. Humans especially children and vegetarians are daily exposed two- to three-fold higher to *Alternaria* toxins according to the European Food Safety Authority (EFSA) assessment of the human dietary exposure of *Alternaria* toxins [31]. *Alternaria* toxins have been investigated at in vitro scale by several research studies over the past decade. However, insufficient data are available about their in vivo hazardous effects [12].

Gastrointestinal organs are thought to be the most liable to harmful alternariol consequences [32]. Homeostasis of oral cavity depends mainly on salivary glands as saliva is responsible for fighting microbes, maintaining pH and carbohydrates catalysis in the mouth [33]. The most common way of Alternariol toxicity is the oral way through food, after which it can be absorbed by enterocytes [24]. The experimental model of oral gavage, utilized in this work, declared the Alternariol intake by food consumption.

Our histopathological examination of Alternariol (AOH) treated group revealed parenchymal disorganization of parotid acini, ultrastructural examination confirmed these results; it demonstrated several forms of degeneration in the serous acinar cells and widening of intercellular spaces with many collagen fibers. Fernández-Blanco et al. [34] and Tiessen et al. [35] attributed cellular degenerative insult of AOH to induction of ROS generation, with a suggested provenance of this ROS production being through AOH metabolism. Burkhardt et al. [36] elucidated this assumption, they documented that AOH undergo aromatic hydroxylation by CYP450 enzymes and phase 1 metabolism enzymes generating both reactive catechols and hydroquinone. It is well-established that such reactive semiquinones and quinones go through a redox cycling process resulting in the generation of ROS [37].

The results of our work provide evidence that AOH induces various cyto-degenerative changes in parotid acini of group III; we reported several vacuoles that appeared in the acinar cells’ cytoplasm that displaced the nuclei peripherally. Ultrastructural picture clarified these results; numerous large cytoplasmic vacuoles, irregularly dilated rough endoplasmic reticulum and whopping electron lucent granules affected acinar cells exposed to AOH treatment. Ambudkar [38] explained these results as ROS induce damage of selective lipid raft domains (LRDs) of plasma membrane including caveolin1 which significantly increases Ca^2+^ influx into acinar cells. In turn, this increases intracellular Na^+^ influx into the cell [39]. Synchronously, the rise in intra-acinar Ca^2+^ regulates the insertion of AQP5 water channels into the apical plasma membrane, thus substantially increasing water tension and distention of rough endoplasmic reticulum “RER” cisternae. Furthermore, the redox cycling process induced by AOH and subsequent ROS generation [40] directly alter acinar lysosomal membranes. Sohar et al. [41] confirmed that disruption of lysosomal membranes favors leakage of damaging lysosomal exoglycosidase, which is synthesized by epithelial cells of salivary ducts.

We observed that AOH induced various pro-inflammatory morphological responses; parotid acini were widely separated by thick connective tissues, congested blood vessels and cellular infiltrations with different inflammatory cells such as mast cells with many secretory granules and plasma cells with heterochromatic nuclei and prominent RER. These findings were in line with Solhaug et al. [42], who reported significant upregulation of inflammatory cytokines TNFα and IL-6 mRNA expression in RAW 264.7 mouse macrophages. Bansal et al. [43] confirmed AOH inflammatory potential in skin; it induced hyperplasia, enhanced prostaglandin E2 and cAMP production side by side with increased COX-2, cyclin D1 and prostanoid EP2 receptor expression in mouse keratinocytes.

Notably, we observed that AOH elicited variable nuclear responses in light microscope slides; some nuclei appeared small and pyknotic, others were actively dividing nuclei. Ultrastructurally, some acinar cells contained large sized nuclei, whereas others had heterochromatic irregular shrunken nuclei. These findings were in line with Schrader et al. [44] and Solhaug et al. [45], who studied AOH effects on nuclear morphology in mouse macrophages; they reported large G_2_ nuclei, few true mitotic cells, several abnormally shaped nuclei, chromatid breaks, kinetochore-negative micronuclei and abnormal Aurora B bridges suggesting interfered cytokinesis, which could also explain abnormally large sized nuclei.

For the past decade, researchers focused mainly on the in vitro mutagenic potentials of AOH. Therefore, we sought to explore the in vivo genotoxic potential of AOH via comet assay. Our results proved alternariol-induced DNA damaging effect in parotid acini, as it revealed a significant increase in percentage of tailed nuclei, tail length, tail DNA % and unit tail moment of nuclei of acinar cells compared with that of the control group. These findings were in accordance with Lehmann et al. [13], who evidenced AOH related inhibition of DNA synthesis and cell proliferation, and Brugger et al. [46], who reported changes in the hypoxanthine-guanine phosphoribosyl transferase (*HPRT*) gene locus in mouse lymphoma cells. Interestingly, DNA damaging events included phosphorylation of histone H2AX and check point kinase-1 in addition to turning on of p53 and subsequent increase of p21 [40]. Several mechanisms are involved in alternariol DNA damaging effects; however, AOH oxidative pathway and interaction with DNA topoisomerase remain the benchmarks [34]. Fleck et al. [47] reported that AOH- induced reactive catechols react covalently with DNA forming depurinating adducts at the N-7 of guanine and the N-3 of adenine. Tiessen et al. [48] added that AOH exposure induced complex distributions of γH2AX histones which are paramount biomarkers of DNA double strand breaks (DSBs) and strong indicator that AOH-induced DSBs are important triggering signals for G2 arrest and autophagy [49]. AOH has been proved as a DNA topoisomerase poison with certain selectivity for its IIa isoform [50]. DNA topoisomerases are mainly required for rejoining of the phosphodiester bonds of DNA strands during the final stages of DNA replication. Somma et al. [51] reported that AOH establishes stabilized covalent topoisomerase–DNA intermediates, besides inhibiting its catalytic activity.

Gallic acid was used in the present study, since earlier research featured its role as an extremely potent natural antioxidant [52]. Our histopathological and ultrastructural results proved considerable restoration of parotid cyto-architecture after gallic acid treatment of AOH-induced changes. Interestingly, we reported significant decrease in percentage of tailed nuclei and other DNA parameters in nuclei of acinar cells after gallic treatment compared with the nuclei of AOH group. The potency of gallic acid as paramount antioxidant owes to three principle features; free radical scavenging activity, maintenance of endogenous antioxidant defense system and prevention of lipid peroxidation. Marino et al. [53] reported GA scavenging efficiency as compared to melatonin, sesamol, protocatechuic acid and capsaicin. In accordance with our results, Reckziegel et al. [54] and Ghaznavi, et al. [55] confirmed GA-induced elevated levels of SOD and CAT, which reported substantial improvement after GA treatment. The observable amelioration of AOH-induced inflammatory cellular infiltrations in GA group suggests its anti-inflammatory properties. These results were in line with Ahn et al. [56] who proved that GA suppressed prostaglandin E^2^ (PGE^2^), TNF-α, IL-1β and NF-κB expression in RAW264.7 macrophages.

4-hydroxynonenal (4-HNE) is α,β-unsaturated hydroxyalkenal generated by peroxidation of n-6 polyunsaturated fatty acid, it is a stable product of lipid peroxidation that acts as a key mediator of oxidative stress-induced cytotoxic effects [57]. We investigated lipid peroxidation status via 4-HNE immunohistochemical expression in parotid acini. We reported significant increase of area percent of 4-HNE expression in AOH treated group in comparison to a control one. Interestingly, MDA levels in parotid tissue homogenates were also substantially elevated. Sadhu et al. [58] explained these results; they claimed that AOH-induced nitric oxide elevation is a key mediator of lipid peroxidation and induced cell death. On the contrary, our results asserted gallic acid mediated significant reduction of both area percent of 4-HNE expression and MDA levels in parotid acini. These results were in line with Akinrinde and Adebiyi [59], who proved GA mediated neuroprotection via reduction of NO and lipid peroxidation products levels in the brain.

Taken together, our data demonstrated alternariol-induced in vivo histopathological and genotoxic alterations on parotid gland. Furthermore, they proved the competency of gallic acid in reversing these effects.

## Figures and Tables

**Table 1:** The area percent of anti-4-HNE immune stained sections in different groups.

Group	Area Percent (Mean ± SD)	ANOVA	LSD POST Hoc Test (Compared to Control Group)
Control group I	4.69 ± 0.43	0.000	
Alternariol group III	15.52 ± 0.74 ^a^		0.000
Gallic + AOH group IV	5.12 ± 0.62 ^b^		0.086

Values are presented as mean ± SD. ^a^ Significant as compared to control. ^b^ Non-significant as compared to control.

**Table 2:** Statistical comparison of Comet assay results in different groups.

Group	COMET % (Mean ± SD)	ANOVA	LSD POST Hoc Test (Compared to Control Group)
Control group I	9.9 ± 2.7	0.000	
Alternariol group III	16.6 ± 5.3 ^a^		0.000
Gallic+ AOH group IV	11.7 ± 4.7 ^b^		0.021
	**TAIL LENGTH (Mean ± SD)**		
Control group I	5.68 ± 1.9	0.000	
Alternariol group III	3.84 ± 0.9 ^a^		0.011
Gallic+ AOH group IV	5.13 ± 1.1 ^b^		0.045
	**TAIL DNA % (Mean ± SD)**		
Control group I	9.51 ± 1.4	0.000	
Alternariol group III	10.58 ± 1.7 ^a^		0.034
Gallic+ AOH group IV	9.65 ± 1.5 ^b^		0.142
	**TAIL MOMENT (Mean ± SD)**		
Control group I	0.48 ± 0.09	0.000	
Alternariol group III	0.62 ± 0.13 ^a^		0.014
Gallic+ AOH group IV	0.51 ± 0.11 ^b^		0.236

Values are presented as mean ± SD. ^a^ Significant as compared to control. ^b^ Non-significant as compared to control.

**Table 3:** Malondialdehyde (MDA), Catalase (CAT) and superoxide dismutase (SOD) and activity in different groups.

Group	MDA (Mean ± SD)	ANOVA	LSD POST Hoc Test (Compared to Control Group)
Control group I	9.1 ± 4.3	0.000	
Alternariol group III	19.3 ± 6.7 ^a^		0.000
Gallic + AOH group IV	10.7 ± 5.4 ^b^		0.042
	**CAT (Mean ± SD)**		
Control group I	0.49 ± 0.03	0.023	
Alternariol group III	0.31 ± 0.01 ^a^		0.012
Gallic+ AOH group IV	0.47 ± 0.02 ^b^		0.134
	**SOD (Mean ± SD)**		
Control group I	22.19 ± 1.12	0.000	
Alternariol group III	9.38 ± 4.59 ^a^		0.000
Gallic+ AOH group IV	20.47 ± 1.19 ^b^		0.078

Values are presented as mean ± SD. ^a^ Significant as compared to control. ^b^ Non-significant as compared to control.

## Abbreviations

AOH	Alternariol
GA	gallic acid
4-HNE	4-hyroxynonenal
MDA	Malondialdehyde
CAT	Catalase
SOD	superoxide dismutase
